# Characterization of the renal phenotype in *RMND1*‐related mitochondrial disease

**DOI:** 10.1002/mgg3.973

**Published:** 2019-09-30

**Authors:** Brian J. Shayota, Nhon T. Le, Nasim Bekheirnia, Jill A. Rosenfeld, Amy C. Goldstein, Michael Moritz, Dennis W. Bartholomew, Matthew T. Pastore, Fan Xia, Christine Eng, Yaping Yang, Dolores J. Lamb, Fernando Scaglia, Michael C. Braun, Mir Reza Bekheirnia

**Affiliations:** ^1^ Texas Children's Hospital Houston TX USA; ^2^ Department of Molecular and Human Genetics Baylor College of Medicine Houston TX USA; ^3^ Baylor College of Medicine Houston TX USA; ^4^ Renal Section Department of Pediatrics Baylor College of Medicine Houston TX USA; ^5^ Department of Pediatrics and Division of Child Neurology University of Pittsburgh School of Medicine Pittsburgh PA USA; ^6^ Department of Pediatrics Division of Nephrology University of Pittsburgh School of Medicine Pittsburgh PA USA; ^7^ Division of Molecular and Human Genetics Nationwide Children's Hospital Columbus OH USA; ^8^ Baylor Genetics Baylor College of Medicine Houston TX USA; ^9^ Department of Urology Weill Cornell Medicine New York NY USA; ^10^ BCM‐CUHK Center of Medical Genetics Prince of Wales Hospital ShaTin Hong Kong SAR

**Keywords:** chronic kidney disease, Mitochondrial disease, renal transplantation, *RMND1*

## Abstract

**Background:**

The nuclear encoded gene *RMND1* (Required for Meiotic Nuclear Division 1 homolog) has recently been linked to RMND1‐related mitochondrial disease (RRMD). This autosomal recessive condition characteristically presents with an infantile‐onset multisystem disease characterized by severe hypotonia, global developmental delay, failure to thrive, sensorineural hearing loss, and lactic acidosis. Renal disease, however, appears to be one of the more prominent features of RRMD, affecting patients at significantly higher numbers compared to other mitochondrial diseases. We report the clinical, histological, and molecular findings of four RRMD patients across three academic institutions with a focus on the renal manifestations.

**Methods:**

Four patients were identified for the purpose of this study, all of whom had molecular confirmation at the time of inclusion, which included the common pathogenic variant c.713A>G (p.N238S) as well as the three rare variants: c.485delC (p.P162fs), c.533C>T (p.T178M), and c.1317 + 1G>C splice donor variant. Medical history and laboratory findings were collected from the medical records and medical providers.

**Results:**

In this study, all four patients developed renal disease characterized as tubulopathy (3/4), renal tubular acidosis (2/4), interstitial nephritis (1/4), and/or end‐stage renal disease (4/4) necessitating renal transplantation (2/4). Histological evaluation of renal biopsy specimens revealed generalized tubular atrophy and on electron microscopy, abundant mitochondria with pleomorphism and abnormal cristae.

**Conclusion:**

Our experience with RRMD demonstrates a specific pattern of renal disease manifestations and clinical course. Patients are unlikely to respond to traditional chronic kidney disease (CKD) treatments, making early diagnosis and consideration of renal transplantation paramount to the management of RRMD.

## Graphical abstract

RMND1‐related mitochondrial disease (RRMD) is a complex mitochondrial condition that has a particularly high risk of severe and early‐onset renal disease compared to other mitochondrial disorders. In this study of four patients, we have shown that the clinical phenotype may include tubulopathy (3/4), renal tubular acidosis (2/4), interstitial nephritis (1/4), and/or end‐stage renal disease (ESRD) necessitating renal transplantation (4/4). Additionally, patients are unlikely to respond to traditional chronic kidney disease (CKD) treatments, making early diagnosis and consideration of renal transplantation paramount to the management of RRMD.

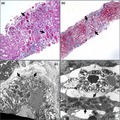

## 1 INTRODUCTION

Mitochondrial diseases commonly present as a complex manifestation of multisystem impairment with a predilection for metabolically active cells. This impairment typically includes varying degrees of neurologic, cardiac, ophthalmologic, and musculoskeletal dysfunction. Historically, renal disease was considered to be a relatively uncommon feature with an estimated 5%–19% of patients experiencing clinically significant renal impairment (Martin‐Hernandez et al., 2005; Rotig & Munnich, 2003). *RMND1*‐related mitochondrial disease (RRMD) represents a unique mitochondrial disease with a particularly high risk for clinically significant renal disease compared to other mitochondrial diseases, affecting approximately 66% of patients (Ng et al., 2016).

Separately, Garcia‐Diaz et al. and Janer et al. first described clinical cases of RRMD in 2012, linking it to biallelic pathogenic variants in the nuclear‐encoded gene *RMND1* (Required for Meiotic Nuclear Division 1 homolog, OMIM 614,917) (Garcia‐Diaz et al., 2012; Janer et al., 2012). *RMND1* belongs to the evolutionary conserved sif2 family of genes that share the DUF155 domain. Additionally, it has three isoforms that contain an N‐terminal mitochondrial localization signal, coiled‐coil region, and C‐terminal transmembrane domain (Garcia‐Diaz et al., 2012). The protein product of *RMND1* is known to have an integral role in the translation of mtDNA‐encoded polypeptides, which is an essential step in forming the subunits of the oxidative phosphorylation complexes. It has been hypothesized to achieve this by serving as an anchor for mitochondrial ribosomes near the site of mRNA maturation, however, the exact mechanism remains unclear (Janer et al., 2015).

Clinically, patients with RRMD typically present with an infantile‐onset multisystem disease characterized by hypotonia, global developmental delay, failure to thrive, leukoencephalopathy with temporal cysts, sensorineural hearing loss, and lactic acidosis (Ng et al., 2016; Ravn, Neland, Wibrand, Duno, & Ostergaard, 2016; Ulrick et al., 2017). The spectrum of renal disease previously described in RRMD patients includes various degrees of chronic kidney disease (CKD), arterial hypertension, dysplastic/hypoplastic kidneys, and electrolyte abnormalities (Ng et al., 2016). Overall, the renal prognosis is poor with many patients progressing to end‐stage renal disease (ESRD) and requiring renal replacement therapy and/or transplantation. In this report, we further define and expand the RRMD phenotype with a focus on the unique pattern of renal involvement.

## 2 PATIENT PRESENTATIONS

Patient 1: This patient presented as a 6 year old female with history of developmental delay, intellectual disability, sensorineural hearing loss, absent speech, and short stature, as well as diminished cerebral white matter volume and leukodystrophy based on MRI imaging. A biopsy of the patient's quadriceps muscle revealed that she had a marked reduction in complex IV activity, suggesting a mitochondrial disorder. The patient's renal disease presented at 6 years of age, which progressed from CKD III at 7 years of age, to CKD IV at 9 years of age, and finally CKD V at 11 years of age at which point she was placed on dialysis and soon after underwent a deceased donor renal allograft. The transplantation was well tolerated and at her most recent evaluation at 14 years of age she remains clinically stable with normal renal allograft function.

Patient 2: This patient presented as a 2‐month‐old female with diarrhea and electrolyte imbalance, originally thought to be secondary to pseudohypoaldosteronism type I. At 8 months of age she was found to have moderate cerebral volume loss with delayed myelination and bilateral sensorineural hearing loss later treated with cochlear implants. Additionally, she had difficult to control hypertension with recurrent hypokalemia, hyponatremia, and acidosis. A renal biopsy revealed chronic tubular interstitial nephritis with extensive tubular atrophy, tubular sclerosis, and a chronic inflammatory infiltrate. Higher magnification on electron microscopy revealed abnormal mitochondria, suggestive of a mitochondrial cytopathy. Mitochondrial respiratory chain enzyme activities from a skin biopsy specimen of the patient were all within the normal range. The patient's renal disease progressed to CKD IV by 5 years of age with the recommendation to undergo renal transplantation, however, she remains stable at 7 years of age.

Patient 3: This patient presented as a 4‐month‐old female infant with global developmental delay, refractory epilepsy, hypotonia, microcephaly, and cortical visual impairment. MRI imaging of the brain revealed cerebral volume loss with white matter abnormalities, basal ganglia calcifications, and temporal cystic changes similar to that observed in congenital cytomegalovirus infections. At 10 years of age, her phenotype evolved to included short stature and intellectual disability. The patient's renal disease has included ESRD with secondary hypertension and renal tubular acidosis. The patient has progressed further into renal failure and has been recommended for renal transplantation at 10 years of age. This patient has been included in prior publications (Parikh et al., 2016), but new information regarding the renal phenotype has been included in this report.

**Figure 1:**
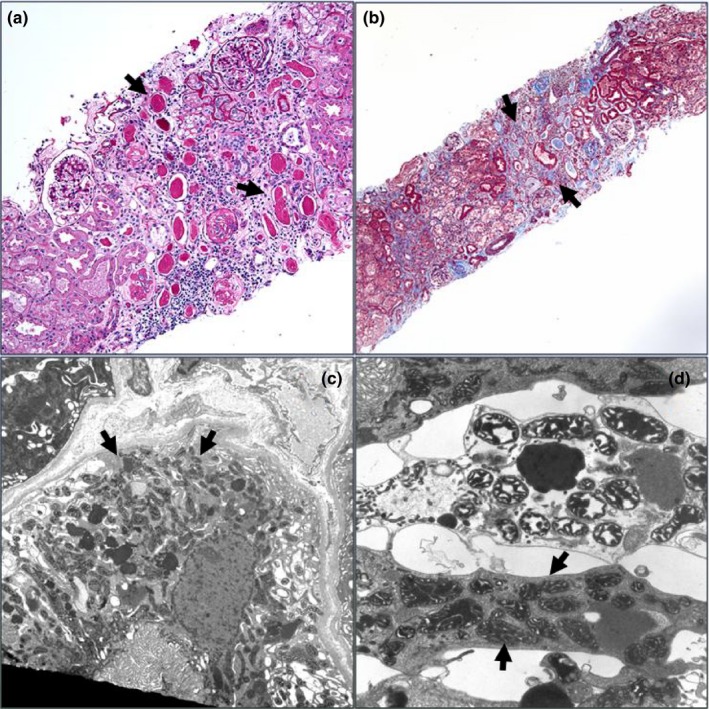
Histology of renal biopsy specimen of patient 4 reveals tubular atrophy consistent with the most common renal phenotype related to RMND1. (a) A PAS stain image showing areas of tubular atrophy with no significant glomerular disease. Note thyroidization of some of the tubules and the interstitial widening (PAS, 20×). (b) A Masson trichrome stain showing interstitial fibrosis and separation of tubules with some interstitial inflammation. (Masson 10×). (c) An electron micrograph showing a low magnification view of tubular epithelial cell with abundant mitochondria (9,080×). (d) A higher magnification image showing some variability in size and shape of mitochondria but no significant inclusions or pathologic changes. (23,700×)

Patient 4: This male patient presented with a history of reported hypoxic ischemic encephalopathy, sensorineural hearing loss, and failure to thrive. In the neonatal period, the patient had multiple cardiac arrests, pulmonary hypertension, and required extracorporeal membrane oxygentation (ECMO) for 8 days. Renal tubular acidosis developed by 6 months of age and by 18 months he rapidly progressed with hyponatremia, hyperkalemia, hyperuricemia, and hypouricosuria that was initially thought to be pseudohypoaldosteronism. A subsequent renal biopsy revealed tubular atrophy with interstitial inflammation and fibrosis. High magnification with electron microscopy found the tubular epithelial cells to have prominent mitochondria with some pleomorphism and broad, thickened cristae without crystalloid inclusions (Figure 1). By 5.5 years of age, the patient had been placed on renal replacement therapy and at 6.5 years of age received a renal transplant. The patient was most recently evaluated at 10 years of age with a well‐functioning transplant. This patient has been included in prior publications (Ng et al., 2016; Parikh et al., 2016), but new information regarding the renal phenotype has been included in this report.

## 3 MOLECULAR TESTING

Molecular testing for confirmation of the diagnosis was made in all four patients (Table 1). The results for the four unrelated probands include the most commonly reported pathogenic variant c.713A>G (p.N238S) as well as the three rare variants: c.485delC (p.P162fs), c.533C>T (p.T178M), and c.1317+1G>T splice donor variant. All three of these less common variants are reported as pathogenic according to the American College of Medical Genetics guidelines (Richards et al., 2015) and have been reported in other patients with the exception of the c.533C>T (p.T178M) variant. However, a different nucleotide change at the same c.533 position that also results in a threonine to lysine substitution has been described in previous reports and labeled as a pathogenic variant as well. This variant had since been reclassified as a pathogenic variant.

**Table 1:** Summary of patients' demographics, genotypes, and phenotypes

	Pt #1	Pt #2	Pt #3	Pt #4
Age (years)	13.9	7.2	7.7	9
Sex	F	F	F	M
*RMND1* nucleotide change	c.485delC c.713A > G	c.533C > T c.713A > G	c.713A > G (homozygous)	c.713A > G c.1317 + G>T
*RMND1* amino acid change	p.P162fs p.N238S	p.T178M p.N238S	p.N238S (homozygous)	p.N238S splice variant
Intellectual disability	**+**	−	**+**	Unk
Global developmental delay	**+**	−	−	**+**
Motor delay	−	**+**	**+**	**+**
Speech delay	**+**	−	**+**	Unk
Microcephaly	−	−	**+**	−
Sensorineural hearing loss	**+**	**+**	**+**	**+**
Cortical vision loss	−	−	**+**	−
Short stature	**+**	−	**+**	+
Brain anomalies	CVL, WMA	CA	BGC, CVL, WMA	Encephalopathy
Cardiac anomalies	−	−	HTN, PFO	LVH
Muscle anomalies	LA, CIVD	−	HPO, HT/S	unk
Renal dysfunction	**+**	**+**	**+**	**+**

Note

The “+” signifies that the patient is positive and “−” negative for a given phenotype. The “unk” signifies it was not ascertained whether or not the phenotype was present at the time of evaluation.

Abbreviations: BGC, basal ganglia calcification; CA, cortical atrophy; CIVD, complex IV deficiency; CVL cerebral volume loss; HT/S, hypertonia/spasticity; HTN, hypertension; LA, lactic acidosis; LVH, left ventricular hypertrophy; PFO, patent foramen ovale; WMA, white matter abnormality

## 4 DISCUSSION

The importance of mitochondrial dysfunction in the pathophysiology of renal disease has gained momentum in recent years. This association is not surprising given that among different organs, the kidney is second only to the heart in mitochondrial count and oxygen consumption (Duann & Lin, 2017). However, not all mitochondrial diseases present with the same renal phenotype. For example, mitochondrial myopathy, encephalopathy, lactic acidosis, and stroke‐like episodes (MELAS) and a select few defects in nuclear encoded genes (*CoQ2*, *CoQ6*, *PDSS2*, and *ADCK4*) that impair coenzyme Q_10_ biosynthesis, classically may present with steroid‐resistant nephrotic syndrome and focal segmental glomerular sclerosis (FSGS) with evidence of podocyte effacement, dysmorphic mitochondria, and arteriolar hyalinosis (Ashraf et al., 2013; Gucer et al., 2005; Seidowsky et al., 2013; Yanagihara, Oyama, Tanaka, Nakaji, & Nishimura, 2001). This finding contrasts with other mitochondrial diseases such as myoclonic epilepsy and ragged red muscle fibers (MERRF), Pearson syndrome, Kearns–Sayre syndrome, GRACILE syndrome, and some types of Leigh syndrome which more commonly develop a proximal tubular defect manifested by loss of electrolytes and presence of low molecular weight proteins in the urine (Che, Yuan, Huang, & Zhang, 2014). The mechanism by which mitochondrial defects can present with such starkly different phenotypes is not well understood at this time. While RRMD has more in common with the latter group of mitochondrial disorders, it is unique in that it presents at such an early stage of the disease and with such a high frequency.

Overall, our patients had a similar clinical presentation of RRMD compared to prior reports with global developmental delay, hypotonia, sensorineural hearing loss, failure to thrive, white matter disease, and renal disease (Table 1). Although two of the patients had been included in prior reports, additional information was obtained to better define the classification and natural history of their renal disease in this report. Notably, all four patients had some degree of renal disease, which is consistent with the prior report with a stated incidence of 66% (Ng et al., 2016). It has also been suggested that the presence of renal disease is a good prognostic factor. However, it seems more likely that those with a more severe disease course leading to death before 12 months of age have potentially not lived long enough to develop renal disease, which does not necessarily always present clinically in the neonatal period. In this study, renal disease had been identified as early as 2 months in patient 2 and as late as 6 years in patient 1.

More specifically the clinical presentation of renal disease in our patients often included electrolyte imbalance secondary to a tubulopathy (3/4) and/or acidosis secondary to renal tubular acidosis (2/4) (Table 2). Interestingly, these clinical findings were often first suspected to be pseudohypoaldosteronism type 1 (3/4), given the similar findings of salt‐wasting renal disease and acidemia. Aldosterone insensitivity had also been suspected in one other patient reported by Janer et al (Janer et al., 2015) before the diagnosis of RRMD had been made. Therefore, RRMD should always be considered in the differential for suspected pseudohypoaldosteronism type 1, especially given the differences in management and prognosis for the two disorders.

**Table 2:** Summary of patients' renal manifestations and the need for kidney transplantation

	This report				Taylor 2014	Janer 2015	Ng 2016†	Gupta 2016	Total
	Pt #1	Pt #2	Pt #3	Pt #4					
Renal dysfunction	**+**	**+**	**+**	**+**	5/6	**+**	20/31	**+**	31/43
Pseudohypoaldosteronism‐like features	Unk	**+**	**+**	**+**	Unk	**+**	Unk	Unk	4/4 reported
Chronic renal failure due to interstitial nephritis	Unk	**+**	−	Unk	Unk	Unk	Unk	**+**	2/3 reported
Tubulopathy	Unk	**+**	**+**	**+**	Unk	**+**	Unk	**+**	5/5 reported
Renal tubular acidosis	Unk	−	**+**	**+**	2/6	Unk	11/35	Unk	15/44 reported
Interstitial inflammation on renal biopsy	Unk	**+**	Unk	**+**	Unk	Unk	Unk	**+**	3/3 reported
Abundant mitochondria in epithelial cells on renal biopsy	Unk	**+**	Unk	**+**	Unk	Unk	Unk	**+**	3/3 reported
End‐stage renal disease	**+**	**+**	**+**	**+**	Unk	Unk	11/35	**+**	16/40 reported
Recommended for renal transplantation	**+**	**+**	**+**	**+**	Unk	Unk	3/35	unk	7/39 reported
Received renal transplantation	**+**	−	−	**+**	Unk	Unk	3/35	Unk	5/39 reported

Note

The “+” signifies that the patient is positive and “−” negative for a given phenotype. The “unk” signifies it was not ascertained whether or not the phenotype was present at the time of evaluation.

^†^ Information regarding one patient included in this report (patient #4) and Ng et al. excluded from totals of Ng et al. to avoid counting the same patient twice.

Microscopic examination of renal biopsies specimens has also been useful in guiding providers to the diagnosis of a mitochondrial disorder, although the changes generally do not appear to be specific to RRMD. Findings from this report include evidence of tubular atrophy with thyroidization, interstitial inflammation, and an over abundance of mitochondria seen on the renal biopsy specimen of patient 4. In another patient reported by Gupta et al, renal biopsy had revealed tubulointerstitial damage with immature glomeruli as well as mildly enlarged mitochondria with a fluffy granular matrix (Gupta et al., 2016). Granular swollen epithelial cells with abundant abnormally shaped mitochondria, in general, have been suggested as a strong indicator of mitochondria‐related kidney disease (Finsterer & Scorza, 2017; Kobayashi, Goto, Nagata, & Yamaguchi, 2010). This appears to hold true for RRMD as well, at least among the few diagnosed patients who have undergone renal biopsy.

In this study, we have also shown that the renal disease seen in RRMD is often progressive and can lead to the need for renal replacement therapy and/or renal transplantation, as observed in all four of our patients. These results suggest that patients with RRMD tend to not respond to other traditional treatments of CKD like proper blood pressure control and a low protein diet, and thus the discussion for renal transplantation should begin early in the disease process. Fortunately, the few reported RRMD patients who have received a renal transplant have had positive outcomes. This includes patients 3 and 4, both of whom had uncomplicated renal transplants performed.

Still, there remains much to be learned about RRMD and the pathophysiological mechanism by which the renal disease occurs with such high frequency compared to other mitochondrial disorders. Although no proven effective treatment for the renal disease seen in mitochondrial disease exists, there are encouraging findings for some specific mitochondrial conditions such as PDSS2‐associated CoQ_10_ deficiency where administration of CoQ_10_ was found to have beneficial effects in affected mice (Saiki et al., 2008; Widmeier et al., 2019). Perhaps in the future new treatment modalities will be developed for other mitochondrial‐related renal diseases such as RRMD to prevent and/or slow the progression of renal dysfunction and delay the need for renal replacement therapy and transplantation.

## 5 CONCLUSION

In summary, we have characterized the clinical and more specifically the renal phenotype observed in RRMD. Our findings suggest that the pattern of renal disease in RRMD can help differentiate it from other causes of pediatric renal disease including other mitochondrial diseases. Furthermore, our experience has shown a gradual progression of renal disease, often leading to the need for renal replacement therapy and transplantation. Therefore, regular monitoring for renal dysfunction and early consideration for renal transplantation should be a mainstay in the management of RRMD affected patients.

## CONFLICTS OF INTEREST

The authors declare that they have no conflict of interest.
